# Steady Decline of HBV DNA Load under NAs in Lymphoma Patients and a Higher Level of qAnti-HBc Predict HBV Reactivation

**DOI:** 10.3390/jcm13010023

**Published:** 2023-12-19

**Authors:** Yiqi Liu, Reyizha Nuersulitan, Chi Zhang, Na Huo, Jun Li, Yuqin Song, Jun Zhu, Weiping Liu, Hong Zhao

**Affiliations:** 1 Department of Infectious Disease, Center for Liver Disease, Peking University First Hospital, No. 8 XishiKu Street, Xicheng District, Beijing 100034, China; eiky-lyq@outlook.com (Y.L.); zhangcdoctor@126.com (C.Z.); huona123456@sohu.com (N.H.); liangliang5398@sina.com (J.L.); 2 Key Laboratory of Carcinogenesis and Translational Research (Ministry of Education), Department of Thoracic Medical Oncology, Peking University Cancer Hospital & Institute, Beijing 100143, China; reyiza1993@126.com; 3 Key Laboratory of Carcinogenesis and Translational Research (Ministry of Education), Department of Lymphoma, Peking University Cancer Hospital & Institute, No. 52 Fucheng Road, Haidian District, Beijing 100143, China; songyuqin@bjmu.edu.cn (Y.S.); zhu-jun2017@outlook.com (J.Z.); 4 Department of Infectious Diseases, Peking University International Hospital, Beijing 102206, China

**Keywords:** lymphoma, diffuse large B-cell, hepatitis B virus infection, HBVr, qAnti-HBc

## Abstract

**Background:** Patients with lymphoma and chronic hepatitis B virus infection need to be treated with both chemotherapy and nucleotide analogue (NA) therapy. However, dynamic changes in HBV DNA loads with increasing chemotherapy cycles are lacking. It is unknown whether HBV replication markers, namely, the quantitative hepatitis B core antibody (qAnti-HBc), HBV RNA, and the hepatitis B virus core-related antigen (HBcrAg), are also markers for predicting HBV reactivation (HBVr). **Methods:** From 29 June 2010 to 6 December 2021, the data of patients with single-site diffuse large B-cell lymphoma and HBV infection (HBsAg+ and HBsAg−/anti-HBc+) were collected from a hospital medical record system, retrospectively. Serum HBV DNA loads (using real-time fluorescent quantitative PCR tests), qAnti-HBc levels (using a newly developed chemiluminescent particle immunoassay), HBV RNA levels (using the simultaneous amplification testing method based on real-time fluorescence detection), and HBcrAg levels (using a Lumipulse G HBcrAg assay) were tested, and factors related to HBVr were analyzed. **Results:** Under NAs, the HBV DNA loads of 69 HBsAg+ lymphoma patients declined from 3.15 (2.13–4.73) lg IU/mL to 1.00 (1.00–1.75) lg IU/mL, and further declined to 1.00 (1.00–1.04) lg IU/mL at the end of a 24-month follow-up. The qAnti-HBc levels decreased gradually during chemotherapy in HBsAg+ lymphoma patients (F = 7.090, *p* = 0.009). The HBV RNA and HBcrAg levels remained stable. A multivariate analysis revealed that higher qAnti-HBc levels (1.97 ± 1.20 vs. 1.12 ± 0.84 lg IU/mL, OR = 6.369, [95% CI: 1.523–26.641], *p* = 0.011) and higher HBV RNA levels (1.00 ± 1.13 vs. 0.37 ± 0.80 lg copies/mL, OR = 3.299, [95% CI: 1.229–8.854], *p* = 0.018) were related to HBVr in HBsAg−/anti-HBc+ lymphoma patients. **Conclusions:** HBV DNA loads declined under NAs during chemotherapy in lymphoma patients. In HBsAg−/anti-HBc+ lymphoma patients, a higher level of baseline serum qAnti-HBc and HBV RNA levels can predict the likelihood of HBVr during chemotherapy.

## 1. Introduction

Hepatitis B virus (HBV) infection is one of the most prevalent health conditions worldwide, with an all-age prevalence and chronic HBV infection rate of 4.1%, affecting around 3.16 million people all over the world [1]. HBV may cause liver damage; the virus optimizes its life cycle to enable long-term persistence in liver tissue by establishing a plasmid-like covalently closed circular DNA (cccDNA) form [2]. Chronic active HBV infection leads to chronic hepatitis B (CHB), which accounts for 30% of all liver cirrhosis death and 40% of hepatocellular carcinoma death [3]. On the other hand, lymphoma is one of the most common malignant tumors in China [4]. The World Health Organization (WHO)’s GLOBOCAN 2020 report revealed 6829 new cases of Hodgkin’s lymphoma (HL) and 92,834 new cases of non-Hodgkin’s lymphoma (NHL) in China in 2020 [5]. Interestingly, people infected with HBV have a two- to three-fold greater risk of developing NHL compared to uninfected people [6]. The mechanism behind this is not so clear, but it is likely due to the hepatotropic and lymphotropic nature of HBV, which assures its replication in lymphoid tissue [7]. However, studies have found that HBV infection is not correlated with HL [8].

Since immunosuppression is presently the mainstay of lymphoma treatment, many lymphoma patients who are coinfected with HBV may experience fluctuating serum HBV DNA loads or even HBV reactivation (HBVr). Furthermore, patients with HBVr may postpone scheduled chemotherapy, or present with abnormal liver function, resulting in adverse effects on lymphoma treatment outcomes. According to the American Association for the Study of Liver Diseases (AASLD), HBVr from anti-cancer therapies occurred in 41% to 53% of HBsAg−positive and anti-HBc–positive patients, and 8% to 18% of HBsAg−negative, anti-HBc–positive patients [9]. In China, the HBV DNA and alanine aminotransferase (ALT) levels of these patients must be monitored during chemotherapy, and prophylactic anti-HBV therapy is suggested for HBsAg–positive patients [10,11].

A systematic review showed that Rituximab (Ritux) can effectively improve the complete remission rate of lymphoma patients [12]. On the other hand, Ritux is an evidence-based drug that can potentially induce HBVr [13]. Therefore, we detected the HBV DNA loads in both HBsAg positive, anti-HBc positive (HBsAg+) lymphoma patients and HBsAg negative, anti-HBc positive (HBsAg−/anti-HBc+) lymphoma patients during the whole chemotherapy cycle (C) and at a 24-month follow-up (M), in order to find out the changes and characteristics of the HBV DNA loads in lymphoma patients during chemotherapy and the follow-up. The new factors are as follows: the quantitative hepatitis B core antibody (qAnti-HBc), produced by Wantai BioPharm, measures the total anti-HBc level (IgM, IgG) using a double-antigen sandwich technique. This was the most widely used immunoassay for anti-HBc quantification [14]. In CHB natural history studies, qAnti-HBc levels in patients during the immune clearance and reactivation phases were significantly higher than those in the immune tolerance and low replication phases [15,16]. HBV RNA and hepatitis B virus core-related antigen (HBcrAg) levels, which have been related to quantities of cccDNA in CHB patients’ liver cells [17,18], were tested in these lymphoma patients every two chemotherapy cycles. The qAnti-HBc levels in these patients were also tested. Dynamic changes were observed along with changes in HBV DNA loads, and since there were several patients with HBVr, the related factors in these patients were also examined.

## 2. Material and Methods

### 2.1. Study Design and Patients

This was a retrospective study. Eligible patients were identified through a hospital medical record system and consented to participate between 29 June 2010 and 6 December 2021 at the Peking University Cancer Hospital. The inclusion criteria were as follows: (1) patients were HBsAg−positive, or HBsAg−negative but anti-HBc-positive; (2) patients had a confirmed diagnosis of diffuse large B-cell lymphoma (DLBCL) from biopsy results; and (3) patients received at least four cycles of immunochemotherapy. Exclusion criteria were as follows: (1) patients had involvement of the central nervous system; and (2) patients had a human immunodeficiency virus or other hepatitis virus coinfection.

The following procedures followed were in accordance with the Helsinki Declaration, approved by The Ethical Committees of Peking University First Hospital (2022Yan284-002). 

### 2.2. Definition of HBV Reactivation (HBVr)

HBVr was defined according to the AASLD guidelines [9]. In HBsAg-positive, anti-HBc–positive patients, it is defined as one of the following: (1) more than (≥) 2 log_10_ (100-fold) increase in HBV DNA compared to the baseline level; (2) HBV DNA ≥ 3 log_10_ (1000) IU/mL in a patient with previously undetectable level (since HBV DNA levels fluctuate); (3) HBV DNA ≥ 4 log_10_ (10,000) IU/mL, if the baseline level is not available. For HBsAg−negative but anti-HBc-positive patients, the following criteria are reasonable for HBVr: (1) HBV DNA is detectable or (2) reverse HBsAg seroconversion occurs (reappearance of HBsAg). A hepatitis flare is reasonably defined as an ALT increase to ≥3 times the baseline level and >100 U/L. 

### 2.3. Data Collection

Blood routine tests, blood biochemistry tests, and HBV DNA were tested in every chemotherapy cycle and every three months after the cessation of chemotherapy. HBV DNA was assayed in Peking University Cancer Hospital using real-time fluorescent quantitative PCR with a detection range of 10 to 10^8^ IU/mL (Northeast Pharm Co., Shenyang, China). Serum qAnti-HBc was measured by a newly developed chemiluminescent particle immunoassay with an upper limit of 100,000 IU/mL (Wantai Co., Xiamen, China) [19,20]. Serum HBV RNA was detected using the RNA simultaneous amplification testing method (HBV-SAT) based on real-time fluorescence detection with an upper limit of 10 [8] copies/mL (Rendu Biotech Inc., Shanghai, China) [21]. Serum HBcrAg was quantified using the Lumipulse G HBcrAg assay and Lumipulse G1200 Analyzer with an upper limit of 10,000 U/mL (Fujirebio, Tokyo, Japan).

### 2.4. Statistical Analysis

Data were reported as mean ± standard deviation (SD, for Gaussian distribution) or median (Q1–Q3, for skewed distribution) for continuous variables and as numbers (percentages) for categorical variables. Chi-square or Fisher’s exact tests (categorical variables), student *t*-test (normal distribution), or Man–Whitney U test (skewed distribution) were used to detect the differences between binary variables. One-way ANOVA and post hoc analysis (Bonferroni) were used to compare the differences of qAnti-HBc/HBV RNA/HBcrAg in different DNA levels. The HBVr-related factors were explored using univariate (*p* < 0.1) and multivariate COX regression. The diagnostic accuracy of markers concerning HBVr was analyzed using receiver operating characteristic curves (ROC) and expressed as the area under the ROC curves (AUROC) and 95% confidence interval (CI). The sensitivity, specificity, positive predictive value (PPV), and negative predictive value (NPV) were calculated. The optimal cut-off values of markers were obtained when Youden’s index was fixed at the maximum value. Spearman’s rank tests were used to analyze the associations between HBV DNA and HBV RNA/HbcrAg. All statistical analyses were performed using SPSS version 26.0 software (SPSS, Inc., Chicago, IL, USA). *p* values less than 0.05 (two-sided) were considered statistically significant.

## 3. Results

### 3.1. Study Population

**Figure 1:**
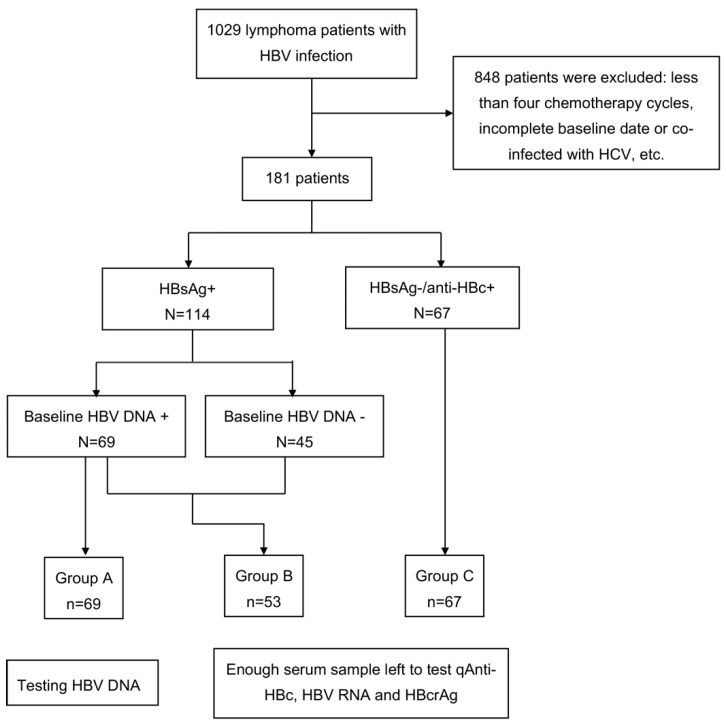
Flow chart of patient enrollment and grouping. Group A are HBsAg+, HBV DNA+ patients with HBV DNA tested at every chemotherapy cycle. We identified these patients to see the dynamic HBV DNA load changes under NAs. Group B includes HBsAg+ patients who have remaining serum samples (after testing for HBV DNA) to test qAnti-HBc, HBV RNA, and HBcrAg. Group C includes HBsAg−/ani-HBc+ patients who have remaining serum samples (after testing for HBV DNA) to test qAnti-HBc, HBV RNA, and HBcrAg.

A total of 1029 patients were screened and 181 patients were enrolled in this study, including 114 HbsAg+ patients and 67 HbsAg−/anti-HBc+ patients. In HbsAg+ patients, 69 patients’ HBV DNA load were higher than 10 IU/mL (Group A). Among them, 53 patients retained paired serum samples before and during chemotherapy (Group B). All 67 HbsAg−/anti-HBc+ patients (Group C) had paired serum samples before and during chemotherapy. The patient’s enrollment flow chart is shown in Figure 1. Among the 114 HbsAg+ patients, they all used Entecavir (ETV) except for two patients who received ETV combined with Adefovir dipivoxil and one patient who received Lamivudine. Among them, 111 received nucleoside analogue drugs (NAs) before chemotherapy, and 92 out of 111 (82.88%) received NA therapy less than one month before chemotherapy. Two patients started NAs at the same time as chemotherapy, and only one HBsAg+ patient started NAs treatment after chemotherapy whose HBV DNA was negative at baseline and added ETV when HBV DNA increased to 2.78 lg IU/mL after three months.

The patients were mainly male in both the HBsAg+ and HBsAg−/anti-HBc+ patients. The average age was 56.6 years old. The subgroup of HBV DNA positive patients (Group A, 51.8 years old) was the youngest compared with the HBV DNA negative subgroup (55.0 years old) and HBsAg−/anti-HBc+ patients (Group C, 62.5 years old), *p* < 0.05. There were only eight cirrhotic patients; all of them were HBsAg+ and half were HBV DNA positive. Liver function, platelet count, and prothrombin time (PT) activity were all comparable between HBsAg+ and HBsAg−/anti-HBc+ patients and subgroups HBV DNA positive and HBV DNA negative. The International Prognostic Index (IPI) score in lymphoma was comparable between HBsAg+ patients and HBsAg−/anti-HBc+ patients, and it was also comparable among the HBsAg+ subgroups (HBV DNA positive and HBV DNA negative). The baseline dosages of Vincristine, Anthracycline, Cyclophosphamide (CTX), and Glucocorticoids (GCs) were all comparable. HbsAg−/anti-HBc+ patients received a much larger dose of Ritux (606 ± 88 mg vs. 485 ± 262 mg, *p* < 0.001) and a much higher percentage of using Ritux at baseline (98.5% vs. 74.6%, *p* < 0.001) than HbsAg+ patients. Detailed baseline characteristics are shown in Table 1.

The serum levels of qAnti-HBc, HBV RNA, and HBcrAg were measured using paired serum samples from patients in Groups B and C collected before and during chemotherapy. At baseline, the levels of qAnti-HBc (3.48 ± 0.84 lg IU/mL), HBV RNA (3.34 (0.00–3.96) lg copies/mL), and HBcrAg (4.27 ± 1.99 lg U/mL) were much higher in Group B patients than that of Group C patients, *p* < 0.001. Detailed baseline of Group B and C patients’ characteristics are shown in Table S1 (Supplementary File).

### 3.2. HBV DNA Load Declined Steadily by NAs in Lymphoma Patients but Declined Less Than Patients without Lymphoma

**Figure 2:**
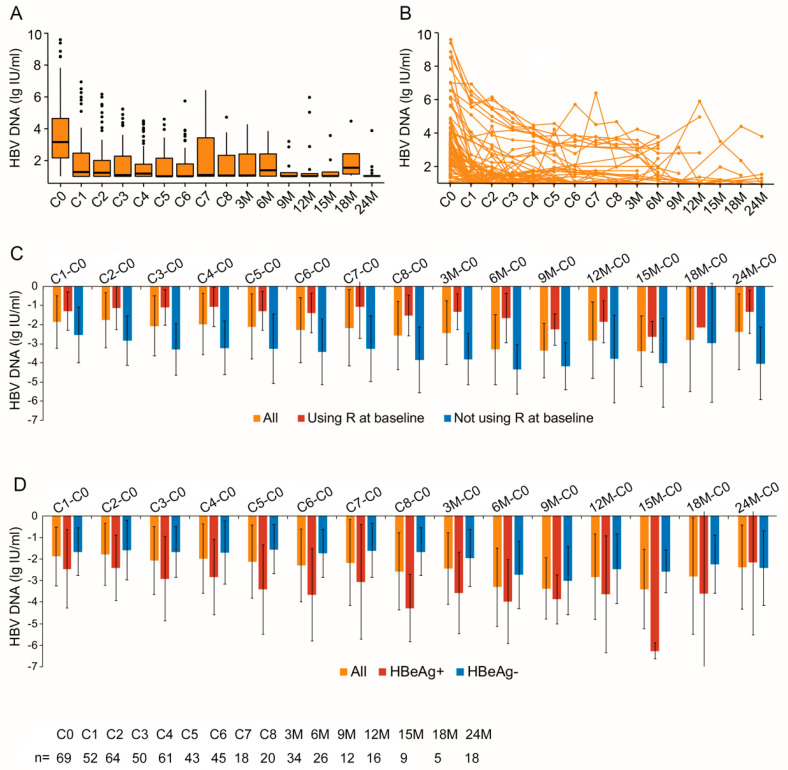
Dynamic decline in HBV DNA load in HBsAg+ lymphoma patients with detectable baseline HBV DNA (Group A). (**A**) The median load of HBV DNA in Group A patients before each chemotherapy cycle (C) and every three months (M) after chemotherapy; (**B**) the HBV DNA load of each patient in Group A before each chemotherapy cycle and follow-up period; (**C**) the decreased load of HBV DNA from baseline (mean) in each cycle of chemotherapy and follow-up period, based on whether the patients used Rituximab (R) at baseline (red color) or not (blue color); (**D**) the decreased load of HBV DNA from baseline (mean) in each cycle of chemotherapy and every three months after chemotherapy, based on whether the patients were HBeAg-positive (red color) or -negative (blue color). The number of patients at each time point is shown at the bottom.

Sixty-nine patients (Group A) were HBV DNA-positive at baseline and were all given ETV; 64 of them were prescribed less than one month before chemotherapy. The serum HBV DNA load decreased steadily by using NAs (F = 13.748, *p* < 0.001), regardless of whether the number of chemotherapy cycles increased. This decline trend persisted throughout the 24-month follow-up period (Figure 2A,B). The load of HBV DNA decreased from 3.15 (2.13–4.73) lg IU/mL at baseline to 1.00 (1.00–1.75) lg IU/mL at the end of chemotherapy and further declined to 1.00 (1.00–1.04) lg IU/mL at the end of the 24-month follow-up.

Twenty-seven patients in Group A underwent chemotherapy without Ritux in the first cycle because of significantly higher HBV DNA loads (5.60 (3.72–7.03) lg IU/mL) than the other 42 patients (2.46 (1.79–3.17) lg IU/m), *p* < 0.001. In patients treated with Ritux in the first cycle of chemotherapy, HBV DNA load still showed a downward trend under the effect of NAs, F = 9.549, *p* = 0.002. However, the decrease in HBV DNA load was less than that of patients who did not use Ritux at baseline (Figure 2C).

At baseline, the HBV DNA load of 16 HBeAg-positive patients (6.77 (4.42–8.54) lg IU/mL) was significantly higher than that of 53 HBeAg negative patients (2.84 (2.04–3.55) lg IU/mL). The load of HBV DNA in HBeAg-positive patients decreased to 3.82 (2.70–4.27) lg IU/mL and 3.36 (2.01–3.76) lg IU/mL after four and eight cycles of chemotherapy. The load of HBV DNA in HBeAg-negative patients decreased to 1.00 (1.00–1.99) lg IU/mL after the first cycle of chemotherapy and was stable with a median load of 1.00 lg IU/mL throughout chemotherapy. Figure 2D shows the decreased values of HBV DNA from baseline. 

We further explored whether chemotherapy drugs and lymphoma could affect the antiviral effect of NAs. Since we had 64 naïve patients who were prescribed ETV just before chemotherapy, we compared the antiviral efficacy of ETV in our patients with patients in the ETV pre-marketing registration trial [22,23]. Unsurprisingly, in the pre-marketing registration trial set of ETV in patients without lymphoma and chemotherapy drug pressure, the decreased load of HBV DNA after 48-week treatment was more significant than in patients with lymphoma, both in HBeAg-positive patients (6.9 ± 2.0 lg IU/mL vs. 3.97 ± 1.94 lg IU/mL, *p* < 0.001) and HBeAg-negative patients (5.0 ± 1.7 lg IU/mL vs. 2.73 ± 1.57 lg IU/mL, *p* < 0.001).

### 3.3. Serum qAnti-HBc Level Decreased Gradually during Chemotherapy in HBsAg-Positive Lymphoma Patients

At baseline, HBsAg-positive lymphoma patients (Group B) had a remarkably higher qAnti-HBc level (3.48 ± 0.84 lg IU/mL) than that of HBsAg−/anti-HBc+ patients (Group C) (1.19 ± 0.90 lg IU/mL), *p* < 0.001.

**Figure 3:**
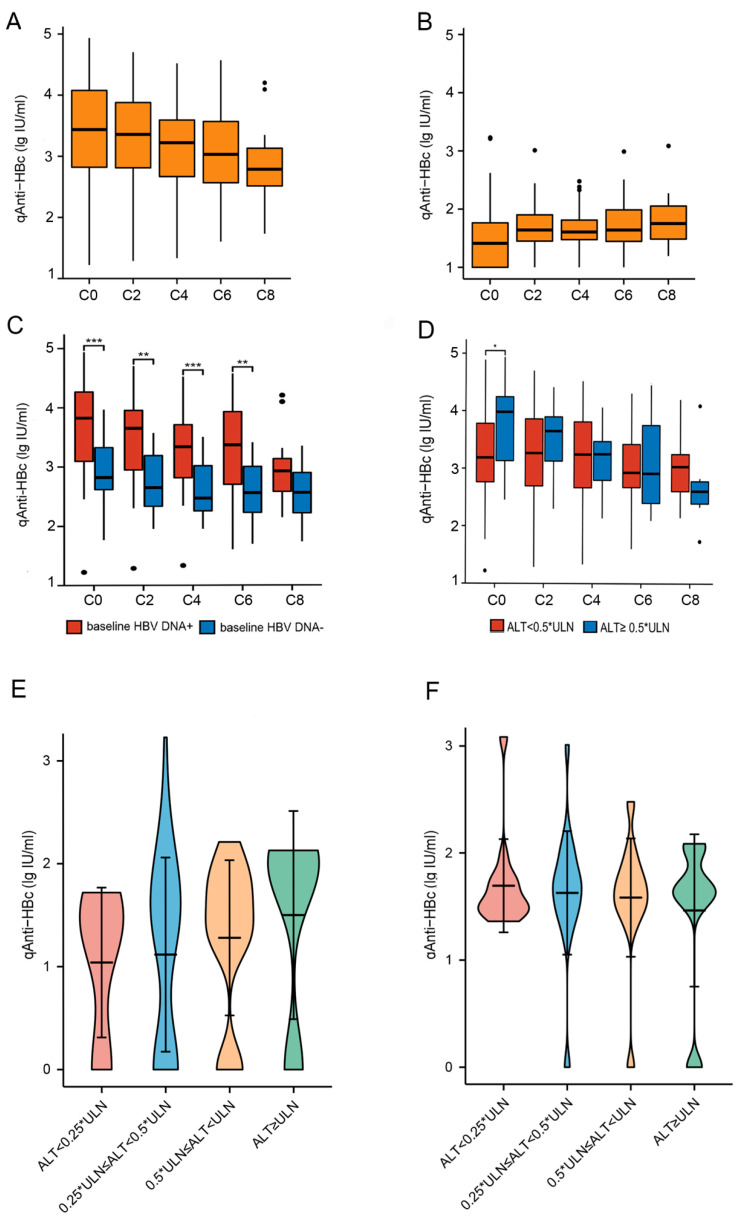
Dynamic changes in serum qAnti-HBc level in lymphoma patients and the relationship between ALT levels in HBsAg+ patients. (**A**) In HBsAg+ patients, serum qAnti-HBc level decreased gradually, no matter whether baseline HBV DNA was detectable ((**C**), red color) or not ((**C**), blue color); (**B**) in HBsAg−/anti-HBc+ patients, serum qAnti-HBc level increased slightly after receiving two cycle chemotherapy and then stabled throughout the chemotherapy; (**C**) described in 3A; (**D**) in HBsAg+ patients, serum qAnti-HBc level remained stable in patients with baseline ALT < 20 IU/L (red color) but decreased gradually in patients with baseline ALT ≥ 20 IU/L (blue color) during chemotherapy; (**E**) in HBsAg+ patients, a synchronous rising trend between qAnti-HBc and ALT was presented (F = 13.723, *p* = 0.001) before chemotherapy when baseline ALT was further divided into four grades; (**F**) in HBsAg+ patients, serum qAnti-HBc level was basically stabled during chemotherapy (the 2nd, 4th, 6th, and 8th cycle) regardless of different grades of ALT. *: *p* < 0.05, **: *p* < 0.01, ***: *p* < 0.001.

For the patients in Group C, the baseline serum qAnti-HBc level was 1.19 ± 0.90 lg IU/mL and the median ALT/AST level was 15/23 IU/L. The serum qAnti-HBc level increased slightly after receiving two cycles of chemotherapy at 1.69 ± 0.40 lg IU/mL and this level remained stable throughout the chemotherapy (Figure 3B). 

In Group B, HBV DNA-positive patients had a much higher qAnti-HBc level than those with HBV DNA undetectable (3.69 ± 0.84 lg IU/mL vs. 2.93 ± 0.55 lg IU/mL, *p* < 0.001). During chemotherapy, serum qAnti-HBc level decreased gradually (F = 7.090, *p* = 0.009) (Figure 3A), no matter whether baseline HBV DNA was detectable or not (Figure 3C). At the end of chemotherapy, 13 patients who were HBV DNA positive turned to negative, and their qAnti-HBc decreased simultaneously from 3.96 ± 0.77 lg IU/mL (baseline) to 3.33 ± 0.71 lg IU/mL (at the end of chemotherapy), *p* < 0.001.

There were 32 patients in Group B with ALT levels lower than 20 U/L (0.5 × ULN) at baseline. Serum qAnti-HBc of these patients (3.29 ± 0.85 lg IU/mL) was significantly lower than patients with ALT ≥ 0.5 × ULN (3.76 ± 0.76 lg IU/mL), *p* = 0.046. While the serum qAnti-HBc level remained at about 3.2 lg IU/mL in patients with baseline ALT < 20 IU/L, the level of qAnti-HBc in patients with baseline ALT > 20 IU/L decreased gradually during chemotherapy (Figure 3D). We further divided the ALT level into four grades and found that the synchronous rising trend between qAnti-HBc and ALT levels was much more clearly presented (F = 13.723, *p* = 0.001) (Figure 3E). During chemotherapy, there were 135 paired ALT levels and qAnti-HBc levels. A stratified analysis showed that the serum qAnti-HBc of different ALT levels was maintained at about 1.5 lg IU/mL during chemotherapy (Figure 3F). 

### 3.4. Serum HBV RNA and HBcrAg Remained Stable under the Chemotherapy

**Figure 4:**
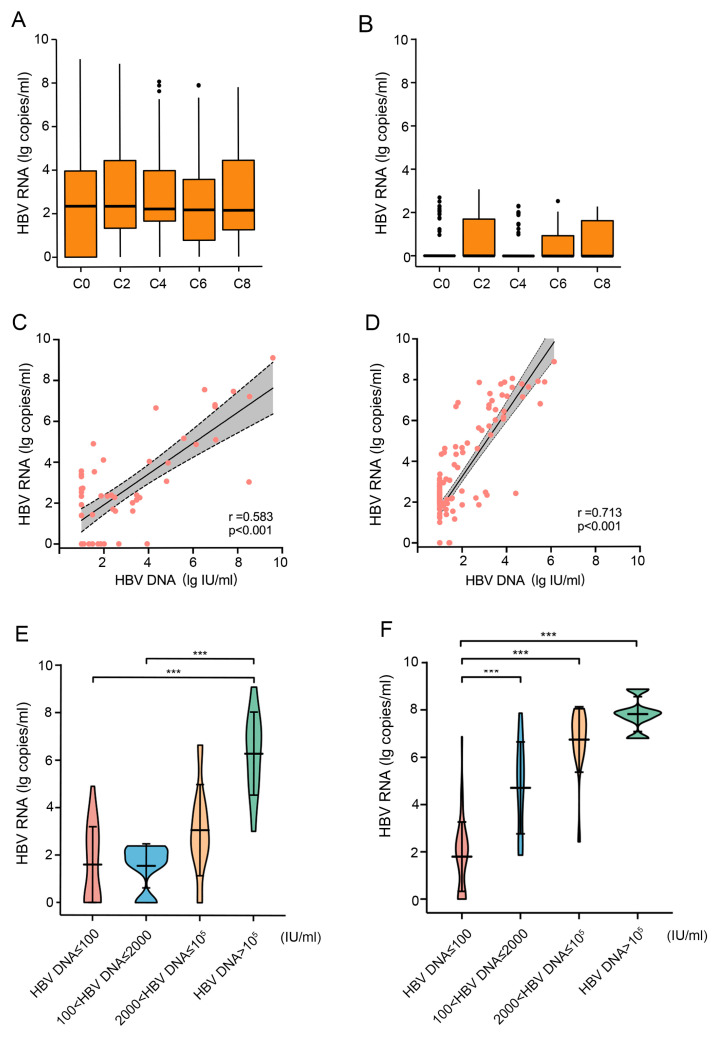
Serum HBV RNA was stable under chemotherapy and positively correlated with HBV DNA load. (**A**) The median HBV RNA level was stable at around 2.20 lg copies/mL in HBsAg+ patients; (**B**) the median HBV RNA level was stable at around 0.00 lg copies/mL in HBsAg−/anti-HBc+ patients; (**C**) in HBsAg+ patients, a positive correlation was found between the level of HBV RNA and HBV DNA before chemotherapy; (**D**) in HBsAg+ patients, a stronger positive correlation was found between the level of HBV RNA and HBV DNA during chemotherapy; (**E**) in HBsAg+ patients, HBV RNA level in the four subgroups before chemotherapy was higher when the grade of HBV DNA was higher: 1.41 (0.00–2.72) lg copies/mL in HBV DNA ≤ 100 IU/mL group, 1.70 (1.61–2.28) lg copies/mL in 100 < HBV DNA ≤ 2000 IU/mL group, 2.72 (2.23–3.98) lg copies/mL in 2000 < HBV DNA ≤ 10^5^ IU/mL group, and 6.76 (2.27–5.11) lg copies/mL in HBV DNA > 10^5^ IU/mL group. But only in HBV DNA ≤ 100 IU/mL group compared with HBV DNA > 10^5^ IU/mL group and 100 < HBV DNA ≤ 2000 IU/mL group compared with HBV DNA > 10^5^ IU/mL group had statistical differences (*p* < 0.001, labeled as ***); (**F**) in HBsAg+ patients, during chemotherapy (the 2nd, 4th, 6th, and 8th cycle), the trend was likely to be followed before chemotherapy, HBV RNA levels were 1.96 (0.00–4.67) lg copies/mL, 4.89 (2.48–5.72) lg copies/mL, 7.15 (6.30–7.69) lg copies/mL, and 7.88 (7.63–7.91) lg copies/mL, respectively. Differences between the three groups were statistically significant (*p* < 0.001, labeled as ***). ***: *p* < 0.001.

The serum HBV RNA level showed no obvious change throughout the chemotherapy. The median HBV RNA level in HBsAg+ patients (Group B) was stable at around 2.20 lg copies/mL (Figure 4A). The baseline HBV RNA level in HBsAg−/anti-HBc+ patients (Group C) was significantly lower (0.00 (0.00, 0.00) lg copies/mL) than that of Group B (2.34 (0.00–3.96) lg copies/mL), *p* < 0.001) and stayed stable during chemotherapy (Figure 4B). The level of HBV RNA and HBV DNA in Group B patients showed a positive correlation, regardless of whether it was before (r = 0.583, *p* < 0.001, Figure 4C) or during (r = 0.713, *p* < 0.001, Figure 4D) chemotherapy. Further analysis showed that the higher the HBV DNA level, the higher the HBV RNA level, regardless of with or without chemotherapy (Figure 4E,F).

Although 13 patients in Group B had undetectable HBV DNA at the end of chemotherapy, their HBV RNA showed no significant change: 1.61 (0.00–2.26) lg copies/mL at baseline and 2.02 (0.00–2.16) lg copies/mL at the end of chemotherapy (*p* = 0.821).

**Figure 5:**
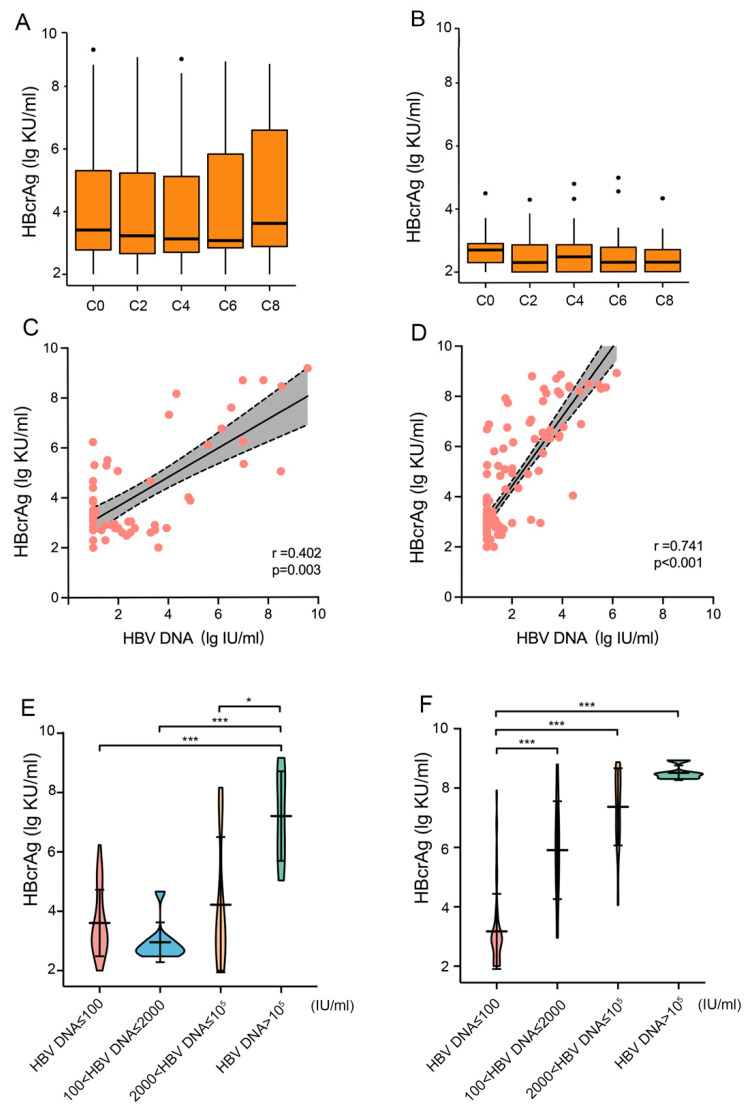
Serum HBcrAg stabled under the chemotherapy and positively correlated with HBV DNA load. (**A**) The HBcrAg level in HBsAg+ patients seems to decline along with the chemotherapy cycle but increased at C8; (**B**) The HBcrAg level in HBsAg−/anti-HBc+ patients stayed stable during chemotherapy; (**C**) in HBsAg+ patients, a positive correlation between HBcrAg and HBV DNA was found before chemotherapy; (**D**) in HBsAg+ patients, a stronger positive correlation between HBcrAg and HBV DNA was found during chemotherapy; (**E**) in HBsAg+ patients, HBV DNA > 10^5^ IU/mL group had the highest HBcrAg level (7.21 ± 1.51 lg U/mL) before chemotherapy compared to HBV DNA ≤ 100 IU/mL group (3.74 ± 1.26 lg U/mL), 100 < HBV DNA ≤ 2000 IU/mL group (2.95 ± 0.67 lg U/mL), and 2000 < HBV DNA ≤ 10^5^ IU/mL group (4.22 ± 2.28 lg U/mL), all *p* < 0.001 (labled as ***); (**F**) in HBsAg+ patients, HBcrAg level in the four subgroups was higher when the grade of HBV DNA was higher during chemotherapy: 3.21 ± 1.32 lg U/mL, 5.91 ± 1.65 lg U/mL, 7.37 ± 1.30 lg U/mL and 8.51 ± 0.25 lg U/mL, respectively. But significant differences were found only between three groups (*p* < 0.001, labeled as ***). *: *p* < 0.05, ***: *p* < 0.001.

The serum HBcrAg level remained stable throughout the study, either in HBsAg+ patients (Group B) (Figure 5A) or Group C (Figure 5B). The level of HBcrAg in Group B positively correlated with HBV DNA, regardless of whether it was before chemotherapy (r = 0.402, *p* < 0.001, Figure 5C) or during (r = 0.741, *p* < 0.001, Figure 5D) chemotherapy. The higher the HBV DNA level, the higher the HBcrAg level, regardless of whether chemotherapy was provided (Figure 5E,F). In the 13 people whose HBV DNA turned undetectable at the end of chemotherapy, their HBcrAg showed no significant change either: 2.85 ± 0.49 lg U/mL at baseline and 2.78 ± 0.42 lg U/mL at the end of chemotherapy (*p* = 0.866).

### 3.5. Higher Baseline Level of qAnti-HBc and HBV RNA Predicted HBVr in HBsAg−/anti-HBc+ Lymphoma Patients

There were ten patients who experienced HBVr: four in HBsAg+ patients (Group B) and six in HBsAg−/anti-HBc+ patients (Group C). HBVr was detected in the period of C2, C2, C2, and C7 (Group B), and C1, C2, C2, C4, C5, and C8 (Group C), respectively. All HBVr patients in Group B received antiviral therapy, but only one patient in Group C received antiviral therapy. Multivariate analysis revealed that a higher qAnti-HBc (1.97 ± 1.20 vs. 1.12 ± 0.84 lg IU/mL, OR = 6.369, [95% CI: 1.523–26.641], *p* = 0.011) and a higher HBV RNA (1.00 ± 1.13 vs. 0.37 ± 0.80 lg copies/mL, OR = 3.299, [95% CI: 1.229–8.854], *p* = 0.018) were related to HBVr in HBsAg−/anti-HBc+ lymphoma patients (Table 2). In HBsAg+ patients, a higher dose of R at baseline (600 ± 82 mg vs. 459 ± 282 mg, *p* = 0.032) was related to HBVr in univariate analysis (Table 2).

The AUROC of qAnti-HBc, HBV RNA, and HBcrAg in Group C patients predicted that HBVr were 0.743, 0.649, and 0.605, respectively (Figure S1, Supplementary File). The cut-off value of qAnti-HBc was 1.60 [95%CI: 0.487–1.000] lg IU/mL. Sensitivity (SE), specificity (SP), positive predictive value (PPV), and negative predictive value (NPV) were 83.3%, 67.2%, 20.0%, and 97.6%, respectively. Other details and details of the three factors in Group B patients are shown in Table 3.

## 4. Discussion

Our study reveals that the serum HBV DNA load decreased steadily by using NAs, regardless of the number of chemotherapy cycles increased, and a declining trend persisted throughout the 24-month follow-up period. HBV DNA load still showed a downward trend under the effect of NAs in patients treated with Ritux from the first cycle of chemotherapy. Serum qAnti-HBc level decreased gradually during chemotherapy in HBsAg positive lymphoma patients. In HBsAg−/anti-HBc+ patients, the serum qAnti-HBc level increased slightly up to 1.69 ± 0.40 lg IU/mL after receiving two cycles of chemotherapy and stayed stable near this level throughout the chemotherapy cycle. Serum HBV RNA and HBcrAg remained stable under chemotherapy treatment. A higher baseline level of qAnti-HBc and HBV RNA predicted HBVr in HBsAg−/anti-HBc+ lymphoma patients.

Studies have shown that qAnti-HBc is related to serum ALT and AST in CHB patients, especially when ALT is within normal range, qAnti-HBc can better reflect the histological inflammation in CHB patients [16,24]. Our research showed a positive correlation between qAnti-HBc and ALT in these CHB patients with lymphoma: qAnti-HBc in the ALT ≥ 0.5 × ULN group was higher than ALT < 0.5 × ULN group (*p* = 0.046), but we did not obtain liver tissue in them. Caviglia, G. P et al. [25] have found that anti-HBc was related to cccDNA, the transcriptional and replicative template of HBV, which may be a useful surrogate for predicting the risk of HBVr. Since some patients experienced HBVr according to AASLD as mentioned above [9], we also compared their demographic, biochemical, and virological indicators. Additionally, we found that a higher qAnti-HBc level at baseline was related to HBVr in HBsAg−/anti-HBc+ patients too (OR = 6.369, [95% CI: 1.523–26.641], *p* = 0.011), with an AUROC of 0.743 (95% CI: 0.487–1.000). This result was consistent with an early study by Yang HC et al. [26] in 2018, who found that high levels of anti-HBc, more than 6.41 IU/mL at baseline, were significantly associated with HBVr (HR = 4.52, [95% CI: 1.75–11.65], *p* = 0.002). Studies have shown that anti-HBs were also related to HBVr, and in Group B, three anti-HBs positive patients did not have HBVr, 50 patients were anti-HBs negative, and four experienced HBVr (0.0% vs. 8.0%, *p* > 0.05). In Group C, there were 52 anti-HB-positive patients with four experienced HBVr, and 15 anti-HBs-negative patients with two experienced HBVr (66.7% vs. 33.3%, *p* > 0.05).

Indeed, for HBsAg+ lymphoma patients, almost all guidelines recommend the same treatment with CHB, while for patients with HBsAg−/anti- HBc+, the guidelines differ slightly. EASL (2017) [27] recommends that prophylaxis should continue for at least 18 months after stopping immunosuppression and monitoring should continue for at least 12 months after prophylaxis withdrawal. AASLD (2018) [9] recommends that once Nas have started, anti-HBV prophylaxis should continue during immunosuppressive therapy and for at least 6 months (or for at least 12 months for patients receiving anti-CD20 therapies) after completion of immunosuppressive therapy. APASL (2021) [28] recommends that the termination of NAs should be considered 6 months after the completion of immunosuppressive therapy. Considering that one patient in our study had HBVr in our last follow-up (24 months), there may be a longer period of antiviral therapy, but it may be our next step to further clarify how long patients should receive NAs.

For the other two HBV virological biomarkers, HBV RNA and HBcrAg, our study found a positive correlation between them and HBV DNA. The correlation index of HBV RNA and HBV DNA at baseline was 0.583, while during chemotherapy, the index was 0.713 (all *p* < 0.001). The correlation index of HbcrAg and HBV DNA was 0.402 and 0.741, respectively (all *p* < 0.001). It is noteworthy to mention that the correlation between the two indicators and HBV DNA has increased during chemotherapy. Additionally, we may need more samples to explain this.

The predominant component of serum HBV RNA is a full-length pregenomic RNA (pgRNA), which is encapsidated by the HBc protein [18]. This component may serve as a possible predictive biomarker to track the safe cessation of antiviral medication. HBV RNA is reportedly related to cccDNA [29], but the specific methods and technical details of serum RNA detection vary widely between different studies [30]. Since we found that the HBV DNA decreased to a negative value at the end of chemotherapy and the HBV RNA and HBcrAg were still positive and had no significant change from baseline, it may suggest that these patients still need to continue antiviral treatment after chemotherapy.

Additionally, Chen EQ et al. [31] reported a positive correlation between HBcrAg levels and liver cccDNA too. In situations where serum HBV DNA levels become undetectable or HBsAg loss is achieved, HBcrAg can still be detectable [32]. Testoni, B et al. [33] proved that HBcrAg is strongly correlated with HBV DNA and cccDNA both in HBeAg+ and HBeAg− patients. Its profile differs drastically in patients in different disease phases, and the level declines with antiviral therapies. Furthermore, one study showed that anti-HBe positive patients with HBVr who underwent long-term NAs treatment and achieved HBsAg loss had detectable HBV RNA at treatment withdrawal, but HBcrAg and HBV DNA were not detected [34].

There are several limitations in our study. Firstly, we did not obtain the liver tissues of these patients, so we could not directly detect the activity of cccDNA in the liver. Secondly, we did not conduct quantitative tests for anti-HBs, which led to a lack of quantitative analysis data on the effect of HBVr. Thirdly, the number of patients in our study was limited, especially those with HBVr, and the ability of qAnti-HBc prediction of HBVr was not verified in the external cohort. Future research should be performed on liver tissue samples and the sample size of HBV-reactivated patients should be expanded.

## 5. Conclusions

In most hepatitis B patients with lymphoma, using nucleoside antiviral drugs can achieve a virological response. However, the risk of hepatitis B reactivation remains in both HBsAg+ and HBsAg−/anti-HBc+ lymphoma patients, especially those whose qAnti-HBc was higher. Therefore, we recommend that patients with hepatitis B and lymphoma, even HBsAg negative, should continue to take oral antivirals during and after chemotherapy for a longer time (at least 24 months).

## Figures and Tables

**Table 1:** Baseline characteristics of all patients.

	Total	HbsAg+				HbsAg−/anti-HBc+	*p* ^2^
		All	Baseline HBV DNA+	Baseline HBV DNA−	*p* ^1^		
N *	181 (120)	114 (53)	69 (38)	45 (15)		67 (67)	
Male, *n* (%)	103 (56.9%)	65 (57.0%)	39 (56.5%)	26 (57.8%)	0.895	38 (56.8%)	0.968
Age, year	56.6 ± 12.6	53.2 ± 12.0	51.9 ± 12.5	55.0 ± 11.4	0.151	62.5 ± 11.5	0.000
BMI, kg/m^2^	23.9 ± 3.9	23.9 ± 4.3	23.9 ± 3.9	24.0 ± 5.1	0.921	23.9 ± 3.2	0.975
Cirrhosis, *n* (%)	8 (4.4%)	8 (7.0%)	4 (5.8%)	4 (8.9%)	0.528	0	0.027
History of HbsAg+, year	2.0 (0.0–20.0)	19.5 (5.0–30.0)	20.0 (10.0–30.0)	15.0 (4.5–20.0)	0.167	0.0 (0.0–0.0)	0.000
HBV DNA, lg IU/mL	1.00 (1.00–1.86)	2.01 (1.00–4.20)	3.30 (1.88–5.74)	1.00 (1.00–1.00)	0.000	1.00 (1.00–1.00)	0.000
PLT, ×10^9^/L	217 ± 85	224 ± 88	215 ± 85	231 ± 90	0.200	203 ± 78	0.113
PTA, %	92.75 ± 16.32	91.28 ± 16.40	90.04 ± 15.73	93.23 ± 17.72	0.328	95.19 ± 16.03	0.131
ALB, g/L	42.2 ± 5.2	42.2 ± 5.6	42.4 ± 5.8	42.2 ± 5.3	0.663	42.2 ± 4.5	0.985
ALT, U/L	16 (12–24)	16 (13–23)	20 (14–26)	13 (11–17)	0.090	15 (11–23)	0.482
AST, U/L	23 (18–27)	23 (18–27)	25 (21–29)	20 (16–24)	0.013	23 (19–27)	0.503
GGT, U/L	23 (17–32)	23 (17–33)	23 (17–37)	23 (17–33)	0.621	22 (16–30)	0.307
ALP, U/L	73.0 (59.0–85.0)	74.5 (58.2–85.5)	75.0 (60.0–86.0)	73.0 (57.0–89.0)	0.614	70.5 (59.0–86.7)	0.964
TbiL, μmol/L	11.7 (9.1–16.6)	12.6 (9.2–17.7)	12.3 (9.1–17.7)	12.7 (9.5–17.6)	0.686	11.3 (9.0–14.9)	0.111
DbiL, μmol/L	3.7 (2.8–4.8)	3.7 (3.1–5.0)	3.9 (3.1–5.9)	3.4 (3.1–4.5)	0.312	3.2 (2.3–4.5)	0.177
HbeAg+, *n* (%)	22 (12.2%)	21 (18.4)	16 (23.2%)	5 (11.1%)	0.139	1 (1.5%)	0.000
qAnti-HBc, lg IU/mL	2.20 ± 1.43	3.48 ± 0.84	3.69 ± 0.84	2.93 ± 0.55	0.000	1.19 ± 0.90	0.000
HBV RNA, lg copies/mL	0.00 (0.00–2.33)	2.34 (0.00–3.96)	2.39 (1.56–4.95)	1.40 (0.00–2.70)	0.004	0.00 (0.00–0.00)	0.000
HbcrAg, lg U/mL	3.38 ± 1.59	4.27 ± 1.99	4.57 ± 2.13	3.50 ± 1.04	0.021	2.67 ± 0.54	0.000
IPI score	1.00 (1.00–3.00)	1.00 (1.00–2.50)	1.00 (0.00–3.00)	1.00 (1.00–3.00)	0.051	1.00 (1.00–3.00)	0.984
First-line chemotherapy cycles	6.3 ± 1.4	6.3 ± 1.4	6.5 ± 1.3	6.0 ± 1.4	0.125	6.2 ± 1.3	0.702
Using Rituximab at baseline, *n* (%)	151 (83.4%)	85 (74.6%)	42 (60.9%)	43 (95.6%)	0.000	66 (98.5%)	0.000
Dose of Rituximab, mg	530 ± 222	485 ± 262	412 ± 296	599 ± 139	0.000	606 ± 88	0.000
Dose of Vincristine, mg	2.8 ± 1.4	2.7 ± 1.4	2.8 ± 1.4	2.6 ± 1.4	0.505	2.8 ± 1.4	0.620
Dose of Anthracycline, mg	67.5 ± 28.8	70.1 ± 25.6	74.3 ± 24.6	63.7 ± 25.9	0.029	63.0 ± 33.3	0.132
Dose of CTX, mg	1182.6 ± 220.18	1203.18 ± 212.54	1207.97 ± 195.71	1195.83 ± 238.19	0.767	1147.59 ± 229.98	0.101
First dose of GCs, mg	50 (0–100)	50 (0–100)	30 (0–100)	60 (30–100)	0.066	60 (30–100)	0.114

All values shown are based on available data. Numeric data are represented as (mean ± SD) or median (upper quartile, lower quartile); *, the number in brackets represents patients with serum sample and tested qAnti-HBc, HBV RNA, and HBcrAg; *p*^1^: *p* value between baseline HBV DNA positive and baseline HBV DNA negative; *p*^2^: *p* value between HBsAg+ and HBsAg−/anti-HBc+ group. Abbreviations: ALB, albumin; ALP, alkaline phosphatase; ALT, alanine aminotransferase; AST, aspartate aminotransferase; BMI, body mass index; CTX: Cyclophosphamide; DbiL, direct bilirubin; GCs, glucocorticoid; GGT, glutamyl transferase; HBcrAg, hepatitis B virus core-related antigen; HBsAg, hepatitis B surface antigen; HBV, hepatitis B virus; IPI score, International Prognostic Index score; PLT, platelet; PTA, prothrombin time activity; qAnti-HBc, quantitative anti-hepatitis B core antigen; TbiL, total bilirubin.

**Table 2:** (a) Univariate and multivariate analysis of HBVr in HBsAg−/anti-HBc+ lymphoma patients. (b) Univariate and multivariate analysis of HBVr in HBsAg+ lymphoma patients.

	Reactivation	Without Reactivation	*p*’-Value	OR (95%CI)	*p*-Value
**(a)**					
N	6	61			
Age, year	61.5 ± 13.4	62.6 ± 11.5	0.824		
HBV DNA, lg IU/mL	1.00 ± 0.00	1.02 ± 0.09	0.682		
ALT, U/L	15 (14–20)	15 (10–24)	0.701		
qAnti-HBc, lg IU/mL	1.97 ± 1.20, 1.89	1.12 ± 0.84, 1.38	0.025	6.369 (1.523–26.641)	0.011
HBV RNA, lg copies/mL	0.86 (0.00–1.94)	0.00 (0.00–0.00)	0.082	3.299 (1.229–8.854)	0.018
HBcrAg, lg U/mL	2.52 ± 0.60, 2.39	2.68 ± 0.54, 2.70	0.492		
IPI score	1.00 (0.50–2.05)	1.00 (0.50–3.00)	0.566		
Using Rituximab at baseline, *n* (%)	6 (100)	60 (98.4)	1.000		
Dose of Rituximab, mg	618 ± 73	605 ± 90	0.739		
Total dose of GCs, mg	215 (45–800)	360 (163–600)	0.926		
ARDI	0.43 ± 0.33	0.73 ± 0.23	0.005		
**(b)**					
N	4	49			
Age, year	55.00 ± 15.06	52.80 ± 12.45	0.738		
HBV DNA, lg IU/mL	2.20 (1.25–3.87)	2.01 (1.00–4.44)	0.603		
HBV DNA > 3.30 lg IU/mL, N (%)	1 (25.0)	17 (34.7)	1.000		
ALT, U/L	16.00 (11.5–184.75)	16.00 (13.00–23.00)	0.430		
qAnti-HBc, lg IU/mL	2.99 ± 1.53, 2.89	3.52 ± 0.77, 3.44	0.225		
HBV RNA, lg copies/mL	3.84 (2.17–6.01)	2.34 (0.00–3.74)	0.259		
HBcrAg, lg KU/mL	5.24 ± 2.14, 4.87	4.19 ± 1.98, 3.34	0.314		
IPI score	2.00 (1.50–2.50)	1.00 (1.00–2.00)	0.452		
Using Rituximab at baseline, *n* (%)	4 (100)	34 (69.4)	0.191		
Dose of Rituximab, mg	600.00 ± 81.65	459.18 ± 282.05	0.032	1.003 (0.996–1.010)	0.388
Total dose of GCs, mg	245 (60.0–400)	300 (180–490)	0.505		

All values shown are based on available data. Numeric data are represented as (mean ± SD) or median (upper quartile, lower quartile); *p*’ value: *p* value of single factor analysis; *p* value: *p* value of multifactor analysis. Abbreviations: ALT, alanine aminotransferase; GCs, glucocorticoid; HBV, hepatitis B virus; HBcrAg, hepatitis B virus core-related antigen; IPI score, International Prognostic Index score; qAnti-HBc, quantitative anti-hepatitis B core antigen.

**Table 3:** Parameters of ROC curve analysis.

	AUROC	95% CI	Cut-off	SE	SP	PPV	NPV
HBsAg+ patients							
qAnti-HBc	0.633	0.190–1.000	2.679	0.500	0.898	0.286	0.957
HBV RNA	0.704	0.471–0.937	3.548	0.750	0.755	0.200	0.074
HBcrAg	0.689	0.469–0.909	4.668	0.750	0.694	0.167	0.971
HBsAg−/anti-HBc+ patients							
qAnti-HBc	0.743	0.487–1.000	1.604	0.833	0.672	0.200	0.976
HBV RNA	0.649	0.422–0.876	1.477	0.500	0.852	0.250	0.945
HBcrAg	0.605	0.334–0.877	2.540	0.667	0.639	0.154	0.951

Abbreviations: AUROC, area under the receiver operating characteristic curve; HBV, hepatitis B virus; HBcrAg, hepatitis B virus core-related antigen; qAnti-HBc, quantitative anti-hepatitis B core antigen; SE, sensitivity; SP, specificity; PPV, positive predictive value; NPV, negative predictive value.

## Data Availability

The data presented in this study are available upon request from the corresponding author.

## Supplementary Materials

The following supporting information can be downloaded at: https://www.mdpi.com/article/10.3390/jcm13010023/s1, Figure S1: The AUROC of qAnti-HBc, HBV RNA, and HBcrAg predict HBVr (A. Group B patients; B. Group C patients).; Table S1: Baseline characteristics of Group B and C patients.
