# Supplementary material for: Steady Decline of HBV DNA Load under NAs in Lymphoma Patients and a Higher Level of qAnti-HBc Predict HBV Reactivation
Source: J Clin Med. 2023 Dec 19;13(1):23. doi: 10.3390/jcm13010023 (PMC10779810; doi:10.3390/jcm13010023)
Supplement: Supplementary file 1 [file jcm-13-00023-s001.zip › jcm-2601752-supplementary.pdf]

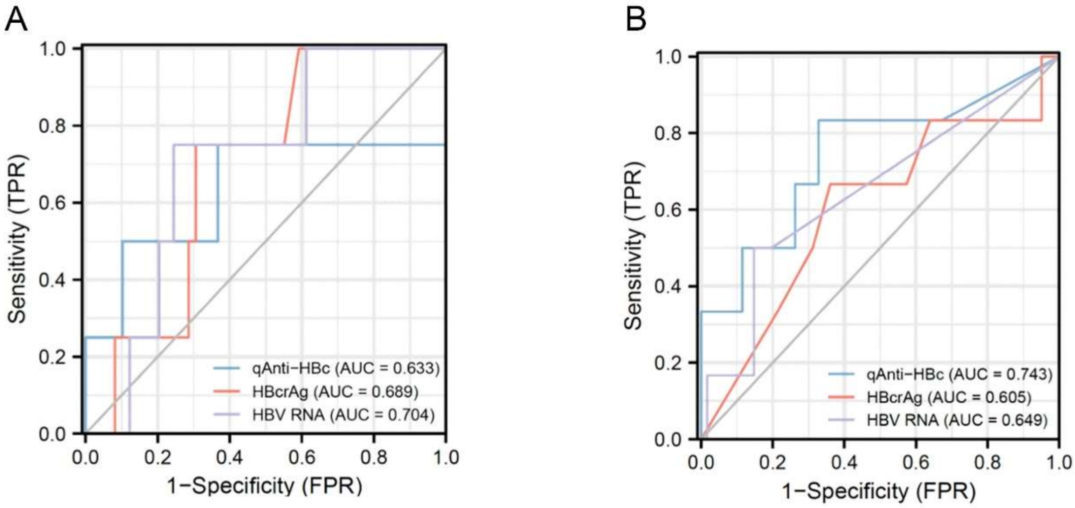

Figure S1: The AUROC of qAnti-HBc, HBV RNA, and HBcrAg predict HBVr (A. Group B patients; B. Group C patients).

Table S1. Baseline characteristics of Group B and C patients.

		HBsAg+				HBsAg-/anti-HBc+	
	Total	all	Baseline HBV DNA+	Baseline HBV DNA-	p^1		p^2
n	120	53	38	15		67	
Male, n (%)	68 (56.7%)	30 (56.6%)	22 (57.9%)	8 (53.3%)	0.768	38 (56.8%)	0.990
Age, year	58.29±12.84	52.96±12.51	51.21±12.90	57.40±10.60	0.105	62.51±11.53	0.000
BMI, kg/m^2	24.09±3.29	24.31±3.35	23.69±3.16	25.89±3.41	0.029	23.91±3.24	0.510
Cirrhosis, n (%)	6 (5.0%)	6 (11.3%)	3 (7.9%)	3 (20.0%)	0.334	0	0.013
History of HBsAg+, year	0.00 (0.00-10.0)	15.0 (1.15-26.0)	7.50 (0.00-0.00)	19.0 (12.0-25.0)	0.084	0.00 (0.00-0.00)	0.000
HBV DNA, lg IU/ml	1.00 (1.00-1.86)	2.16 (1.00-4.86)	3.39 (2.00-6.44)	1.00 (1.00-1.00)	0.000	1.00 (1.00-1.00)	0.000
PLT, ×10^9/L	204.21±79.36	205.13±81.66	203.92±75.17	208.20±99.19	0.866	203.49±78.11	0.911
PTA, %	93.96±16.82	92.39±17.82	92.88±17.16	91.15±20.04	0.761	95.19±16.03	0.383
ALB, g/L	42.63±4.42	43.14±4.34	43.29±4.29	42.75±4.60	0.691	42.23±4.46	0.267
ALT, U/L	16.0 (12.0-23.0)	16.0 (13.0-23.0)	19.0 (13.3-19.0)	14.0 (11.0-15.5)	0.262	15.0 (11.0-22.7)	0.444
AST, U/L	23.5 (18.0-27.0)	24.0 (17.5-28.5)	25.0 (20.5-29.8)	20.0 (16.0-23.5)	0.147	23.0 (19.0-27.0)	0.307
GGT, U/L	23.0 (17.0-31.8)	24.0 (17.0-37.0)	25.0 (19.5-39.0)	24.0 (16.0-31.5)	0.074	22.0 (16.0-30.0)	0.280
ALP, U/L	70.5 (59.0-83.8)	68.0 (57.5-81.5)	71.5 (60.5-81.7)	63.0 (56.5-80.0)	0.336	70.5 (59.0-86.7)	0.778
TBiL, μmol/L	11.8 (9.00-16.5)	13.1 (9.33-18.3)	12.1 (8.53-17.6)	15.1 (12.1-20.0)	0.609	11.3 (9.00-14.9)	0.054
DBiL, μmol/L	3.50 (2.70-4.80)	3.70 (2.90-5.55)	3.80 (2.80-5.50)	3.30 (3.15-5.95)	0.795	3.20 (2.32-4.50)	0.099
HBeAg+, n (%)	15 (12.5%)	14 (26.4%)	13 (34.2%)	1 (6.7%)	0.080	1 (1.5%)	0.000
qAnti-HBc, lg IU/ml	2.20±1.43	3.48±0.84	3.69±0.84	2.93±0.55	0.000	1.19±0.90	0.000
HBV RNA, lg copies/ml	0.00 (0.00-2.33)	2.34 (0.00-3.96)	2.39 (1.56-4.95)	1.40 (0.00-2.70)	0.004	0.00 (0.00-0.00)	0.000

HBcrAg, lg KU/ml	3.38±1.59	4.27±1.99	4.57±2.13	3.50±1.04	0.021	2.67±0.54	0.000
IPI score	1.00 (1.00-2.75)	1.00 (1.00-2.00)	1.00 (0.25-2.00)	2.00 (1.00-3.00)	0.094	1.00 (1.00-3.00)	0.792
First-line chemotherapy cycles	6.26±1.38	6.32±1.44	6.37±1.44	6.20±1.47	0.705	6.21±1.34	0.662
Using Rituximab at baseline, n (%)	104 (86.7%)	38 (71.4%)	23 (60.5%)	15 (100.0%)	0.005	66 (98.5%)	0.000
Dose of Rituximab, mg	545.88±204.52	469.81±274.27	407.89±301.69	626.67±45.77	0.008	606.06±88.45	0.000
Dose of Vincristine, mg	2.98±1.48	3.19±1.59	3.10±1.47	3.42±1.88	0.514	2.82±1.38	0.178
Dose of Anthracycline, mg	66.96±30.71	72.04±26.48	77.11±25.14	59.20±26.21	0.025	62.95±33.33	0.099
Dose of CTX, mg	1161.19±242.41	1178.40±258.46	1181.97±226.04	1169.33±336.10	0.874	1147.59±229.98	0.492
First dose of GCs, mg	50.0 (30.0-100)	50.0 (30.0-100)	30.0 (0.00-100)	100 (100-50.0)	0.056	60.0 (30.0-100)	0.539

All values shown are based on available data. Numeric data are represented as (mean±SD) or median (upper quartile-lower quartile); p^1: p value between baseline HBV DNA positive and baseline HBV DNA negative; p^2: p value between HBsAg+ group and HBsAg-/anti-HBc+ group.

Abbreviations: ALB, albumin; ALP, alkaline phosphatase; ALT, alanine aminotransferase; AST, aspartate aminotransferase; BMI, body mass index; CTX: Cyclophosphamide; DBil, direct bilirubin; GCs, glucocorticoid; GGT, glutamyl transferase; HBcAb, Hepatitis B core antibody; HBcrAg, hepatitis B virus core-related antigen; HBsAg, hepatitis B surface antigen; HBV, hepatitis B virus; IPI score, International Prognostic Index score; PLT, platelet; PTA, prothrombin time activity; qAnti-HBc, quantitative anti-hepatitis B core antigen; TBil, total bilirubin.
